# Educating Students About Digital Health Research Ethics: Curricula Review and Expert Interview Study

**DOI:** 10.2196/82861

**Published:** 2026-03-26

**Authors:** Yier Zhu, Brian McInnis, Tanya Punater, Brittany York, Camille Nebeker

**Affiliations:** 1 Herbert Wertheim School of Public Health and Human Longevity Science University of California San Diego La Jolla, CA United States; 2 The Design Lab University of California San Diego La Jolla, CA United States; 3 Qualcomm Institute University of California San Diego La Jolla, CA United States

**Keywords:** digital health research, ethics education, practice-based learning, qualitative research, research ethics, research training, sociotechnical health systems

## Abstract

**Background:**

The rapid growth of digital health research, involving wearable devices, mobile apps, and sociotechnical health systems, raises complex ethical, legal, and social considerations. While institutional review boards and research ethics frameworks address some concerns, less is known about how students and trainees in digital health are systematically educated to recognize and navigate these challenges. Understanding the scope and content of ethics training is critical to ensuring the responsible development and application of digital health technologies.

**Objective:**

This study investigated how college students are trained to identify and address ethical considerations in digital health research through an analysis of formal curricula and expert perspectives.

**Methods:**

Researchers reviewed 132 syllabi from 76 academic programs across 62 universities and conducted semistructured interviews with 6 leading digital health scholars. All syllabi were coded for instructional content and learning objectives. Researchers conducted open coding and collaboratively applied affinity diagramming to organize the data into hierarchical themes.

**Results:**

All syllabi included instructional content, and most included explicit learning objectives. Analysis identified 7 key themes, which captured both explicit knowledge imparted through formal instruction and tacit knowledge cultivated through laboratory work, mentorship, and applied experiences. Findings highlighted gaps between formal ethics instruction and the realities of research practice.

**Conclusions:**

Ethics education in digital health research develops through the interplay of formal coursework and practice-based training, each fostering complementary skills needed for data-intensive and collaborative environments. Together, these pathways support students in identifying ethical issues, applying principles contextually, anticipating emerging risks, and communicating across disciplines; however, access to experiential learning opportunities remains inconsistent. Strengthening ethics training will require expanding structured early research engagement, cultivating communities of practice, and translating tacit ethical reasoning into accessible resources. Integrating ethical reflection into routine research activities may better prepare future digital health researchers to responsibly design and govern sociotechnical health systems.

## Introduction

As digital technologies become integral to health research and clinical care, students must be prepared to examine related ethical, social, and organizational implications. Digital health tools, from mobile apps and wearables to artificial intelligence (AI) systems, can improve care; yet, their implementation often outpaces understanding of effects on patients, providers, and health systems [1,2]. Our research focuses on digital health research ethics as a sociotechnical concern: technologies both shape and are shaped by institutional contexts, stakeholder relationships, and data infrastructures to coordinate people, groups, and computing resources with a goal of improving patient health and well-being [3,4]. For example, a conversational agent located in a patient’s room could be used to take notes during a clinical visit [5], notify nursing teams when the patient is experiencing increased anxiety [6], and converse with the patient to reduce their feelings of social isolation [7]. Digital health tools, like wearable sensors, offer new ways to monitor patients, both in and outside of the clinic, which have important ethical implications [8]. By adopting a sociotechnical lens, we attend to system components (ie, technologies, data practices, and institutional policies) and social interactions between team workflows and patient/participant engagement to examine emerging ethical issues, including unanticipated privacy and data management risks.

Academic education and training programs play a critical role in preparing digital health researchers and clinicians to navigate ethical challenges like algorithmic bias, data privacy, and access inequities, yet little empirical work has examined the availability and scope of such programs across organizations contributing to digital health research [6]. Unlike general research ethics, digital health research includes characteristics that can strain traditional academic ethics models. These differences include streams of continuous, granular individual-level data that may remain identifiable and blurred lines between research and industry data flows [9-12]. Importantly, informed consent communications are challenged by difficulties explaining passive sensing and other pervasive technologies, data management practices, and evolving algorithms [13-15].

Researchers learn to recognize and mitigate ethical challenges through university courses, workshops, and readings (explicit knowledge) as well as through firsthand opportunities to learn by training with expert research practitioners and working with clinicians, caregivers, and digital health technology designers in the field (tacit knowledge). As digital health ethical and social implications relate to the design and deployment of technology within social, cultural, and organizational contexts, it is critical for students to learn through both explicit coursework and experience-based learning (tacit knowledge). However, less is known about the specific components of this education among disciplines involved in the design and testing of digital health tools. Explicit training supplies common concepts and language; experiential learning develops judgment for context-specific decisions as teams design, deploy, and evaluate sociotechnical systems in health settings [10,11,16]. Because these sociotechnical properties scale through computing infrastructures, the stakes and failure modes in digital health differ from those typically addressed in general research ethics training.

To examine how students gain ethical competencies in digital health research, we analyzed university syllabi to characterize explicit knowledge conveyed through coursework and conducted interviews with leading digital health researchers to understand how tacit knowledge is conveyed through the conduct of research, which can include decisions about consent, data protection, stakeholder engagement, and dissemination [17,18]. Our goals for this study were to (1) evaluate how programs that contribute to digital health research socialize students to ethical and responsible research practices through coursework and (2) describe how expert researchers convey tacit ethical practices to trainees through hands-on training. We do not test hypotheses; rather, this exploratory, mixed evidence synthesis (syllabi and interviews) maps explicit and tacit learning opportunities in digital health research ethics.

## Methods

### Overview

This study used a multimethod qualitative design, integrating 2 complementary components to examine how ethical competencies in digital health research are taught and learned. We analyzed course syllabi to characterize explicit ethics instruction and conducted semistructured interviews with experienced digital health researchers to understand how tacit ethical knowledge is conveyed through research practice. We used the sociotechnical framework to integrate insights from syllabi (explicit knowledge) and interviews (tacit knowledge), examining how instructional content aligned with or diverged from the ethical challenges researchers encounter in sociotechnical research environments. These 2 data sources provide a comprehensive synthesis of explicit and experiential learning.

### Ethical Considerations

The research activities involved minimal risk to participants, and the protocol was reviewed and verified as exempt under 45 CFR 46.104(d)2 by the institutional review board (IRB) at the University of California San Diego (exemption verification 210941). All interview participants received recruitment materials and provided verbal informed consent prior to audio recording. Participants did not receive compensation. Reporting followed the Standards for Reporting Qualitative Research (SRQR), identified by the EQUATOR (Enhancing the Quality and Transparency of Health Research) Network.

Audio recordings and transcripts were stored in University of California San Diego’s secure Microsoft Teams environment, which provides encrypted, access‑restricted cloud storage; only authorized study personnel with institutional credentials could access study files to ensure participant privacy and confidentiality.

### Study Design

The study used 2 complementary components to examine how ethical competencies in digital health research are taught and learned. First, a syllabi analysis was conducted to characterize explicit ethics instruction delivered through university coursework. Second, expert interviews were conducted to examine tacit ethics knowledge conveyed through hands-on research practice. Although designed and analyzed independently, the 2 components together highlight complementary dimensions of ethics education in sociotechnical digital health research. To identify academic programs and researchers relevant to digital health ethics training, this study drew upon a preliminary literature review of sociotechnical digital health research in computing and information science venues. This review informed the selection of academic units for the syllabi search as well as the identification of researchers eligible for the interview component.

### Course Syllabi Collection—Explicit Knowledge

Institutional websites and departmental materials were examined to determine whether units associated with the publications included in the systematic literature review (forthcoming) offered formal or informal training opportunities focused on ethical and responsible research practices. These included required ethics coursework, elective offerings, workshops, journal clubs, and other structured learning activities relevant to sociotechnical systems and digital health research.

A total of 132 course syllabi were collected from academic units identified through the search strategy. Of these, 98 syllabi contained learning objectives and were therefore included in the learning objective analysis. The remaining syllabi were excluded because they lacked learning objectives or did not provide sufficient detail to determine ethics-related instructional content. The final analytic set represents 64 academic programs from universities in the United States and internationally.

When course information was not publicly available, research staff contacted instructors or administrative personnel to request syllabi, course descriptions, and information about relevant instructional activities. Syllabi served as the primary data source for examining explicit ethics-related knowledge conveyed through coursework.

Criteria for identifying ethics-related courses were derived from the syllabi analysis protocol, which specified how the research team determined whether a course contained substantial ethics content. Courses were classified as ethics-focused when the title, description, learning outcomes, or listed topics indicated a primary emphasis on ethical issues relevant to sociotechnical systems that contribute to digital health research. Courses in which ethics was addressed only briefly within a broader technical curriculum were not classified as ethics-focused. When syllabi were unavailable, these criteria were applied to publicly accessible catalog descriptions and course topic summaries.

To determine who to contact within each academic program, research staff reviewed departmental web pages, faculty directories, graduate program administrator listings, and instructional staff profiles to identify individuals most likely to have knowledge of course offerings. Depending on the structure of each department, emails were directed to course instructors, department chairs, program coordinators, graduate advisors, or general departmental accounts.

Beginning on August 18, 2023, and concluding on December 8, 2023, research staff sent up to 4 email communications to relevant instructors and administrative staff to solicit course information. The communication provided an overview of the study and requested (1) syllabi for targeted courses, (2) course descriptions or lists of required graduate courses, and (3) information about formal and informal training opportunities, such as workshops, seminars, journal clubs, or other instructional activities. Recipients were also invited to forward the request to colleagues who might have access to course materials or additional information.

### Syllabi Analysis

After collecting the course syllabi, the content was analyzed to explore the breadth of learning objectives and course topics related to digital health research ethics. Learning objectives, also known as learning goals or outcomes, are statements that define what students should learn or be able to do after completing a course or activity. Course topics refer to the sequence of subject areas covered during a course, often presented along with a schedule for the course lectures and assignments or summarized in a course description. To analyze the collection of course syllabi, our study applied an approach similar to recent studies of courses in technology ethics [17] and social computing [18]. Specifically, 3 researchers reviewed each syllabus to identify learning objectives and course topics. Initial coding was conducted independently by multiple members of the research team, followed by regular consensus-based meetings to review interpretations, resolve discrepancies, and refine analytic decisions. To identify themes, the research team applied an affinity diagramming method [19] to qualitatively organize the learning objectives and course topics into hierarchical clusters. Members of the research team met twice per week for 6 weeks using FigJam (Figma Inc) [20] to support collaborative synthesis. The resulting themes characterize explicit ethics-related knowledge conveyed through coursework and are later interpreted alongside interview findings.

### Interview Recruitment and Procedures—Tacit Knowledge

Semistructured interviews were conducted between August 17 and September 7, 2023. Recruitment involved outreach to a pool of 32 eligible individuals, with up to 3 contact attempts per person. Recruitment emails summarized the study purpose and the general topical domains from which participants could select areas they felt most prepared to discuss. Authors were eligible to participate if they were corresponding or lead authors on included publications and could speak to training, ethical decision-making, and research team practices in digital health research. A total of 6 individuals consented to participate and completed the interview. All interviews were analyzed. Together, the 6 interviewees had 516 publications and 85,252 citations, reflecting their standing in the field.

Eligible researchers received an email invitation describing the study purpose, interview procedures, and plans to audio-record the session. Interviews were conducted via Zoom (Zoom Communications) by a trained graduate student researcher, with a supervising team member present to support consistency in procedures. Verbal informed consent was obtained at the beginning of each interview. All interviews were audio-recorded with participant consent and transcribed using Zoom’s built-in transcription feature.

The semistructured interview guide was informed by our prior literature review and publication-specific analyses of sociotechnical health research practices (forthcoming). Questions were organized around the following five themes identified in our prior analyses: (1) prototyping designs, including how research teams collaboratively prototype systems, protocols, and materials; (2) engaging stakeholders, including how stakeholders are involved throughout the research life cycle; (3) developing informed consent procedures and data protections, including how teams design consent processes and data safeguards; (4) mentoring research, including the role of mentorship in training and guiding research practices; and (5) sharing research, including how research teams disseminate findings and return results to participants and stakeholders. The guide included open-ended prompts within each theme to elicit concrete examples from participants’ experiences. Experts were invited to select two to three themes they felt most prepared to discuss.

### Interview Transcripts Analysis

An iterative, inductive thematic approach was used to analyze interview transcripts. Although the analysis was inductive, interpretation was informed by a digital health research ethics perspective, which sensitized the team to ethical considerations arising from the design and use of engineering-based digital health systems within research and institutional contexts. Following each interview, researchers drafted analytic memos documenting preliminary impressions, emerging concepts, and notable patterns [21]. These memos and associated transcript excerpts were reviewed by supervising researchers to ensure accuracy, deepen interpretation, and maintain analytic rigor.

Building on this initial memoing process, 1 researcher conducted detailed inductive coding to identify recurring concepts, and a second researcher independently reviewed these summaries and coded transcript segments to refine and validate emerging interpretations. The student interviewers and supervising researchers met regularly to discuss analytic decisions, resolve discrepancies through consensus, and iteratively refine the thematic structure; four researchers contributed to the overall analysis. Because the full set of interviews represented a small sample of senior digital health researchers, the team analyzed all available data and refined themes until shared conceptual coherence was reached through regular consensus meetings. Topics from the interview guide are summarized with example prompts in Textbox 1.

**Interview focus areas and example prompts.**

**Ethics-related research practices discussed during interviews**

- Prototyping technology for health research
- Engaging with community stakeholders
- Developing informed consent procedures and data protection policies
- Sharing research data and findings

**Reflective prompts about field development and ethics priorities**

- How do you find and connect with other researchers in your field?
- Where do you look for funding for your research?
- How do you see this research field evolving over the next few years?
- What do you see as key research ethics topics to include in training programs?
- Who sets the guidelines and standards for ethics training in your field?

## Results

### Overview

Across our analysis of 98 course syllabi and 6 expert interviews, we identified seven themes that characterize how ethical competencies in digital health research are taught and learned: (1) broad understanding of ethical principles, (2) applied ethics, (3) research in context, (4) ethics in technology design, (5) anticipating the future, (6) communication practice, and (7) professional responsibility. Some conceptual overlap across themes occurs across communication, team collaboration, and professional responsibility. This is expected given the sociotechnical context of digital health research. Ethical competence is developed and demonstrated across interpersonal interaction, team workflows, and organizational norms, as these are interdependent domains. The sections that follow describe the programs along with each theme and illustrate how explicit and tacit learning opportunities manifest across programs and research environments.

### Academic Programs

The academic programs identified through the search reflect a breadth of disciplines. A majority (n=48, 77%) of the universities represented by the syllabus collection are located within the United States, with 14 (22%) located internationally. The 132 courses include graduate-level instruction for doctoral and medical school students as well as students in a range of master’s degree programs (Table 1). Courses were open to undergraduate and graduate students (n=24, 18%), with the majority (n=64, 48%) limited to undergraduate enrollment.

**Table 1:** Total courses by academic discipline based on the name of the university department.

Academic discipline	Count, n
Bioengineering	2
Biology	2
Cognitive science	2
Communication	2
Computer science	62
Digital media	8
Education	1
Engineering	10
Global health	1
Human-centered computing	3
Industrial design	1
Informatics	18
Information science	11
Innovation	1
Nursing	1
Pediatrics	1
Philosophy	1
Public policy	1
Sociology	1
Total	132

### Sample Size

We analyzed 98 syllabi that included learning objectives to identify explicit ethics-related learning opportunities and identified 7 themes, as summarized in Table 2. Within the 98 syllabi analyzed, 5 universities provided instruction for 5 of the 7 themes, whereas 12 universities incorporated only 1 (19%) theme. None of the academic programs offered a course series that encompassed all 7 learning-objective themes. The following sections describe each theme. Additional examples of learning objectives and theme-specific references are provided in Multimedia Appendix 1.

**Table 2:** Descriptions of themes with the number of learning objectives and courses represented in each theme.

Theme descriptions		Objectives, n	Courses (n=98), n (%)
**Theme 1: broad understanding. Gain a broad understanding of ethical principles and guidelines [22-25]**			
	Learn to apply ethical principles in research, in general	20	18 (18.4)
	Explore how ethical principles have been applied in different contexts	33	18 (18.4)
	Summary	53	31 (31.6)
**Theme 2: applied ethics. Practice applying ethical principles to real-world cases and scenarios [26-29]**			
	Identify health informatics issues from various perspectives	12	4 (4.1)
	Identify ethical challenges related to technology	42	27 (27.6)
	Identify ethical considerations related to privacy and security	17	13 (13.3)
	Learn about ethical practices in data science	28	19 (19.4)
	Summary	99	43 (43.9)
**Theme 3: research in context. Build research skills necessary to investigate ethical questions within specific context [30-33]**			
	Explain uses of AI^a^	19	7 (7.1)
	Learn about data science methods	23	13 (13.3)
	Describe security and privacy processes	37	13 (13.3)
	Develop research and analysis skills	33	16 (16.3)
	Summary	112	42 (42.9)
**Theme 4: ethics in technology design. Learn how ethical principles can play into decisions about human-computer interaction [34-37]**			
	Develop foundational skills in the design process	28	16 (16.3)
	Gain skills in human-computer interaction design	18	6 (6.1)
	Learn about ethical design principles	19	7 (7.1)
	Learn about the design of digital health systems	16	5 (5.1)
	Summary	81	27 (27.6)
**Theme 5: anticipating the future. Learn how to identify and assess ethical issues tied to emerging and futuristic technologies [38-46]**			
	Explore diverse perspectives in technology	15	11 (11.2)
	Explore the societal impact of health technology	14	7 (7.1)
	Explore the societal impact of novel and future technology	24	21 (21.4)
	Explore ELSIs^b^ of technology	36	16 (16.3)
	Introduced to an overview of computing research	33	19 (19.4)
	Gain basic knowledge in business	1	1 (1)
	Summary	123	50 (51)
**Theme 6: communication practice. Develop skills to communicate ethical concerns in group discussions, presentations, and written reports [47-51]**			
	Develop professional communication skills	26	18 (18.4)
	Learn about engaging with diverse audiences	22	18 (18.4)
	Developing critical thinking and reasoning skills	17	13 (13.3)
	Summary	65	42 (42.9)
**Theme 7: professional responsibility. Understand the ethical and professional standards expected of researchers conducting research in the public interest [52-56]**			
	Describe ethical and professional responsibilities in computing	17	14 (14.3)
	Learn about professional ethics in workplaces	11	7 (7.1)
	Develop teamwork and team management skills	13	10 (10.2)
	Summary	41	28 (29)

^a^AI: artificial intelligence.

^b^ELSI: ethical, legal, and social implications.

### Theme 1: Broad Understanding. Principles and Guidelines (eg, Bioethics and Human Rights)

This theme captures how courses orient students to ethical traditions (bioethics, research ethics, and rights-based perspectives) and institutional guardrails (eg, IRB training). The emphasis is on mastering shared vocabulary and analytic lenses that later anchor applied judgments in digital health contexts.

Our analysis found that a third of all courses (31/98) include learning objectives that emphasize training in diverse philosophical approaches to ethical thinking (Table 2, Theme 1). As summarized in Table 2 (Theme 1), foundational objectives appear across a substantial subset of courses but vary in depth from survey-style introductions to more rigorous treatments that require analytic writing and IRB certification. Programs rarely sequence these foundations with later, hands-on components, creating discontinuities that reappear in downstream themes. One course articulates this by stating that students will “develop a working understanding of major traditions of philosophy and ethical theory to provide foundational tools of analysis.” (C108)

Foundational principles serve as a grounding reference point for decisions about informed consent, risk assessment, and mitigation, and expectations for fairness as digital health systems are implemented in new settings. Differences in depth point to gaps in scaffolding from foundational principles to applied and design contexts. These foundational principles matter because they establish the ethical vocabulary and conceptual grounding students later rely upon when making context-specific decisions in data-intensive health environments. Without this grounding, students may struggle to recognize how abstract principles translate into concrete ethical challenges that emerge in sociotechnical systems.

### Theme 2: Applied Ethics. Practice Applying Ethical Principles to Real-World Sociotechnical Contexts

Courses in this theme move beyond abstraction and ask students to apply ethical principles to concrete cases involving algorithmic decision-making, data privacy and security, and responsible AI deployment in health contexts. Applied ethics is positioned as a bridge between frameworks and situated decisions made during design, analysis, and deployment, with related learning objectives appearing in 43 (44%) of 98 courses. These courses emphasized students' ability to analyze ethical challenges (ie, information storage, exchange, security, and privacy) across various contexts, such as health informatics, data science, and computer science (Table 2, Theme 2). For example, “Apply the philosophies and theories covered to computer science problems and scenarios, including risks associated with large language models and algorithmic decision-making” (C55). As shown in Table 2 (Theme 2), applied ethics objectives are prevalent yet unevenly embedded. Some courses integrate iterative casework and debate across the term; others contain a single module within broader technical instruction. Assignments commonly require students to reason through value trade-offs across stakeholders and technical constraints, making ethical salience visible at practical decision points.

Applied ethics training equips students to recognize and navigate tensions where harms materialize, such as in data curation, model evaluation, and deployment pipelines. This applied focus is essential in digital health because ethical tensions often arise at the level of implementation, such as privacy trade-offs in data pipelines or fairness issues in algorithmic modeling. These courses help students identify where harms may materialize and how to reason through competing stakeholder values, trade‑offs in data pipelines, or fairness issues in algorithmic modeling.

### Theme 3: Research in Context. Methods to Study Sociotechnical Interactions

This theme reflects the pairing of technical content (AI/machine learning, security, and data science) with research design and analysis (literature reviews, proposal writing, and qualitative/quantitative reasoning) so students can empirically study sociotechnical systems in health. The focus is not coding per se, but the methodological capacity to investigate ethical questions in context. Results reveal 43% (42/98) of our course syllabi (Table 2, Theme 3) depict broad coverage of research skills objectives. We found that some syllabi emphasize technical fluency, while others center on social scientific inquiry. Few programs explicitly connect these skill sets to institutional realities (eg, governance and clinical workflows), a linkage that becomes crucial in later themes. As an example, C66 states, “This course covers the fundamental principles of the scientific method, and the core skills of planning, designing, executing, evaluating, and presenting research.”

Blending computing and social science methods reveals how risks emerge from interactions across data, tools, institutions, and people; however, explicit integration of where risks and benefits can accrue across clinical and organizational constraints appears inconsistent. Syllabi linked to this theme aim to develop competence by enabling students to investigate how risks, biases, and unintended consequences occur, reflecting the sociotechnical nature of digital health systems. Understanding context is critical for anticipating where ethical safeguards may fail.

### Theme 4: Ethics in Technology Design. Learn How Ethical Principles Can Play Into Decisions About Human-Computer Interaction

Courses link values (accessibility, equity, and sustainability) to concrete design choices for digital health systems. Here, ethics is framed as an up-front design requirement, which may include incorporating inclusive methods, accessibility standards, and sensitivity to domain-specific issues such as digital biomarkers or health disparities, rather than a post hoc audit. One effective way to bridge the gap between ethical principles in theory and the practice of system design is through the incorporation of human-centered design (HCD) and human-computer interaction concepts. This includes examining “how digital health technologies can be designed to achieve health equity [with a] central focus on applying human-centered design concepts to the domain of digital health equity” (C19). Our analysis found that 27% (n=26) of the courses teach students HCD processes (Table 2, Theme 4). Unlike Theme 3, courses in Theme 4 prioritize understanding system design concepts, though systems are also often incorporated into specific research methods.

Introducing criteria into design processes that increase awareness of ethical practices can help teams to operationalize what respect for persons and justice look like in the research design phase. Programs that connect HCD/human-computer interaction to clinical governance appear better positioned to align design intent with downstream demonstration of ethical practices.

#### Explicit and Tacit Knowledge About Research Ethics Through Curriculum and Hands-on Training

As described in the Methods section, digital health research experts, selected based on their prior citation frequency and prominence within the digital health research community, were interviewed to gather their perspectives on the digital health field and to speculate about the ethics training students will need in the future. Quotes from the interviews are uniquely identified by code, P1-P6, with each code corresponding to a specific expert. Because the interview data aligned closely with Themes 5-7, the following theme descriptions integrate evidence from both course syllabi and experts’ perspectives.

### Theme 5: Anticipating the Future. Learn How to Identify and Assess Ethical Issues Tied to Emerging and Futuristic Technologies

Courses in Theme 5 ask students to anticipate ethical, legal, and social trade-offs of emerging or speculative technologies before systems are deployed, with attention to governance, unintended consequences, and positionality. As summarized in Table 2 (Theme 5) , this theme appears in 50 (51%) of 98 syllabi, making it the most prevalent in the corpus; yet, programs vary in how anticipation is taught. Some emphasize legal and policy analysis, others use scenario debates and stakeholder role-play to surface value trade-offs. One course, for example, requires students to “propose recommendations...that represent the perspectives of stakeholders to develop a resolution.” (C2).

Interview accounts show how this anticipatory work becomes a tacit judgment in practice. Researchers described teaching students to reason through downstream risks under uncertainty , particularly around data practices that are easy to overlook but consequential for participants. For instance, with passive sensing of GPS data, students are asked to grapple with consent memory and continuous collection: “it gets turned on, and it runs...continues to pull those data until the person uninstalls the app.... How do you manage that?” (P2). Teams use these moments to clarify what to do when participants later realize some data feel too sensitive to be shared, and to revisit data sharing procedures so that participants are aware of protections throughout a study.

Senior researchers also coach students to communicate the salience of risk when peers view data as “just data.” As one expert noted, “Students might ask, ‘This is just data; why are you making it so hard to access?’ I bring examples of how location data alone can be very compromising or reveal intimate parts of someone’s life.... It can have serious consequences if used beyond consent.” (P6). Another expert emphasized that even deidentified datasets can create reidentification risk when clinical features are unique, requiring thoughtful protection while retaining research value (P4). In parallel, experts voiced concern that AI/large language model adoption in health research is moving faster than frameworks and training, urging explicit conversations about safe, responsible use. As one explained, “we should...figure out ways to talk about how to use these technologies in a safe, ethical, and responsible way.” (P6).

Anticipatory ethics is particularly important in digital health, where emerging technologies outpace regulation and policy. Learning objectives that aim to prepare students to spot plausible harms and governance gaps before deployment, but curricula differ in modality and depth. Interview evidence shows how mentors transform anticipation into on-the-ground judgment, teaching students to (1) notice subtle data risks (eg, passive GPS), (2) communicate why those risks matter to participant welfare, and (3) revisit data management choices as technologies and contexts evolve.

### Theme 6: Communication Practice. Develop Skills to Communicate Ethical Concerns in Group Discussions, Presentations, and Written Reports

Theme 6 centers on the individual’s capacity to surface and reason about ethical issues through argumentation, critique, writing, and public speaking. As shown in Table 2 (Theme 6), communication-focused learning objectives appear in 42 (43%) of the 98 syllabi, but their sophistication varies. This spans from basic presentation mechanics to rigorous argumentation that engages counterpositions with evidence and adapts messages to different audiences.

Many courses build these competencies by having students read extensively, write analytically, and lead structured discussions. One syllabus puts it plainly: students should “read a lot, write a fair amount, and become more comfortable participating in oral discussions and giving an oral presentation.” (C49). Another emphasizes the standard to which this participation is held: “articulate your ethical beliefs precisely and persuasively.” (C116). Together, these objectives position rhetoric as the mechanism by which ethical reasoning becomes public, contestable, and actionable in research settings.

Communication is foundational in ethical practice. Ethical issues in digital health typically surface through dialogue ; these syllabi describe tools to practice talking about ethical challenges. The interview narratives show those tools at work explaining risk, negotiating disagreement, and recording decisions so teams remain accountable to participants and stakeholders.

### Theme 7: Professional Responsibility. Understand the Ethical and Professional Standards Expected of Researchers

This theme focuses on organization-level norms, including professional codes, role clarity, escalation paths for ethical concerns, cross-disciplinary collaboration, and stewardship responsibilities for managing volumes of individual-level sensitive data. As indicated in Table 2 (Theme 7), such objectives appear in 28 (29%) of 98 syllabi, less frequently than communication or anticipation themes. Syllabi in this theme ask students to analyze professional association policies and to consider how responsibilities translate into daily practice in research teams and health organizations; for example, “What special responsibilities do we have as computing professionals? How can we apply the Software Engineering Code of Ethics and ACM Code of Ethics in our daily practice?” (C21).

Tacit practices, described by experts, make the organizational mechanics of responsibility concrete. Senior researchers emphasized modeling norms and setting expectations that are then diffused through laboratories and professional communities. For example, “we have to show that this is the way we do the work.” (P5). Experts also highlighted the need to work across disciplines and with stakeholders (eg, clinicians, patients, and community partners) so that design decisions reflect health system realities and community values; collaboration remains essential but complex, requiring time, care, and clear alignment with funder constraints (P4 and P1).

Experts train laboratory members to conduct stakeholder-engaged digital health research, which includes building relationships, listening across perspectives, and giving back to communities. These are competencies that extend professional responsibility beyond the laboratory into the settings where digital health systems are designed and implemented (P4 and P5).

Professional responsibility is enacted through governance structures and norms (codes, roles, and processes) and through collaboration with diverse stakeholders. Courses introduce these expectations; interviews show how senior researchers make governance visible in day-to-day work, translating ethical intent into accountable team practices.

## Discussion

### Principal Findings

Digital health research operates within complex sociotechnical systems [57], making ethical competence foundational to responsible innovation. This study examined how research ethics education prepares trainees for the sociotechnical realities of digital health research. Findings indicate that ethical competence develops through the interaction of classroom instruction and experiential learning; yet, gaps remain in translating abstract principles into research practice. Addressing these gaps will require instructional strategies that more intentionally integrate ethics training with the collaborative, data-intensive environments in which digital health research occurs.

Research ethics education in digital health relies on both explicit instruction and tacit learning. Classroom teaching introduces guiding ethical principles, analytic tools, and case studies [58], while participation on research teams enables students to identify, discuss, and resolve ethical issues as they arise [59]. Experts in our study described mentoring trainees through decisions affecting participant data privacy, allowing students to observe how choices related to data storage, sharing, and stewardship unfold across a study. These complementary experiences reinforce the reciprocal relationship between teaching and research practice.

Our thematic results illustrate how ethical competence in digital health research emerges through sociotechnical interactions rather than individual reasoning alone. For example, the “applied ethics” and “research in context” themes show how ethical issues arise within data pipelines, modeling practices, and clinical workflows, where technical and organizational factors intersect. Themes such as “communication practice” and “professional responsibility” highlight how interpersonal coordination, role clarity, and governance structures shape ethical action within research teams. Our results confirm that ethics education must prepare trainees to navigate these interconnected sociotechnical dynamics rather than isolated ethical dilemmas.

### Ethics Education as a Sociotechnical Process

Our findings reveal a persistent sociotechnical tension in ethics education. Coursework often emphasizes individual reasoning and abstract principles, whereas ethical challenges in digital health research are collective and infrastructural, emerging through team workflows, data pipelines, institutional requirements, and evolving stakeholder expectations. Ethical competence, therefore, extends beyond cognitive skill to a situated practice shaped by organizational routines, technical affordances, and supervisory norms [60]. Senior researchers play an important role by modeling everyday ethical decision-making and making these processes visible, enabling norms to diffuse across laboratories and professional communities [61]. Cross-training activities, such as journal clubs, further support this process by cultivating a shared vocabulary for reasoning about risks, roles, and constraints.

Because digital health research frequently involves health care systems and community partners, stakeholder communication and team science skills are prioritized in both coursework and experiential training [62]. Experts reported connecting trainees with community collaborators to build practical experience working across sectors, while classroom exercises in perspective-taking and public speaking reinforce these competencies. Such preparation is critical for sustaining multipartner research, where navigating disagreement is often central to maintaining trust.

Despite these complementary pathways, reliance on experiential learning raises concerns about consistency in training. Ethical norms are frequently transmitted within research groups, meaning that uneven access to hands-on sociotechnical decision-making could reinforce inequities in students’ ability to develop ethical judgment [63]. Our syllabus analysis supports this interpretation in that courses commonly address privacy, fairness, and professional responsibilities; however, fewer examine how these principles are enacted within algorithmic pipelines, wearable sensing systems, or clinical governance processes. This gap helps explain why experts view experiential learning as essential for translating abstract principles into practice while underscoring the need for more intentional instructional supports.

### Expanding Experiential Learning Through Instructional Infrastructure

One strategy for expanding access to practice-based learning is to scaffold undergraduate participation through a structured critique modeled after the ethics and society review [64]. Students who have completed research ethics coursework could contribute to proposal review in bounded, time-limited ways. Although ethics and society review relies on expert reviewers and may be difficult to scale, prior work in human-computer interaction demonstrates that scaffolding approaches, such as design critique and historical analysis, help novice learners complete complex evaluative tasks while strengthening ethical reasoning [59,65,66]. Instructors could adopt similar workflows by training students to collaboratively analyze case studies [67]. However, implementation challenges remain, including limited institutional support, uneven student preparedness for complex sociotechnical contexts, and the risk that simplified workflows may obscure ethical tensions. Addressing these constraints will require continued evaluation and the development of shared educational resources supported by professional associations and research sponsors.

### Translating Tacit Insights Into Shareable Resources

Beyond scaffolding, our findings suggest the importance of translating practice-based insight into reusable instructional materials. Tacit knowledge develops through experience and is often conveyed as intuition, making it difficult to formalize. Yet ethical concerns that emerge during research offer opportunities for documentation. Existing tools, such as ethics checklists (eg, the Digital Health Checklist [68]) and design artifacts such as card decks that embed ethical principles into computing and AI research [69,70] demonstrate how abstract guidance can be externalized into prompts that support reflection in practice.

Building on this approach, researchers could create brief ethics discussion prompts when salient concerns arise during a study. Drawing on Parker’s framework [71], such prompts would include a problem statement, a description of the solution space, and a guiding question, accompanied by reflection on how the issue was addressed. Unlike traditional case studies, which often isolate single decisions, these prompts could capture ethical judgment as it unfolds within research workflows. When shared alongside research outputs, they have the potential to convert tacit experience into concise, transferable instructional resources. Adoption, however, raises important questions about documenting sensitive issues, ownership of materials, and incentives for participation, which create tensions that must be addressed to normalize reflective ethical practice in digital health research.

### Theoretical Implications for Digital Health Research

Our findings reconnect research ethics training to sociotechnical theory by illustrating that ethical competence emerges through the interaction of data infrastructures, research workflows, and institutional expectations rather than through formal instruction alone. Experts described guiding trainees through real-time decisions about passive data collection, such as GPS and sensor-based monitoring, where ethical judgment developed in relation to both continuous data flows and governance requirements surrounding consent and stewardship. Viewing ethics education through a sociotechnical lens shifts the focus from individual responsibility toward the systems and environments that shape ethical action. By foregrounding ethics education as a sociotechnical phenomenon, this study extends prior work that frames ethical decision-making as an individual competency. Instead, our findings suggest that ethical capacity is distributed across training environments, research infrastructures, and supervisory practices, underscoring the importance of designing educational systems that support ethical action.

### Implications for Research Ethics Training in Digital Health

Responding to calls for more practice-oriented ethics training in digital health, this study makes 3 primary contributions. First, it provides a thematic analysis of explicit ethics instruction across 98 syllabi representing 76 international academic programs, offering one of the most comprehensive mappings of current curricular approaches. Second, it advances understanding of how trainees develop ethical competence by identifying experiential pathways through which tacit knowledge is cultivated in research settings. Third, it proposes actionable strategies for strengthening instructional infrastructure, including closer integration of classroom learning with research practice, scaffolded opportunities for student participation, educator-researcher learning communities, and the use of embedded ethics discussion prompts to support learning reflective practice.

Collectively, our findings position research ethics education not simply as a pedagogical challenge but as an instructional infrastructure issue within digital health research. Ethical competence is shaped by the environments in which researchers are trained, suggesting that strengthening this infrastructure requires closer integration of classroom learning with structured experiential opportunities, expanded scaffolded participation in ethical review, and mechanisms for translating practice-based insight into shared instructional resources. Advancing these efforts could promote more consistent training, reduce inequities in access to experiential learning, and better prepare the next generation of researchers to navigate the sociotechnical complexities of data-intensive health research.

### Limitations

This study has several limitations related to our sampling strategy. Digital health remains an emerging sociotechnical field with few formal degree programs and no standardized course titles (eg, “digital health research ethics”), making traditional catalog searches impractical. We therefore identified researchers and affiliated programs through Association for Computing Machinery publications to center the study on active research environments; however, this approach may have excluded relevant curricula at teaching-focused colleges and medical schools that prioritize instruction over research. Our sample also reflects a geographic concentration in the United States and Europe, introducing a potential Western bias. Teaching priorities and approaches to ethics education may differ globally, highlighting the need for more internationally representative research. Additionally, syllabi provide insight into intended instruction but do not capture how courses are experienced by instructors or students. Although interviews with senior researchers offered perspectives on mentoring and training, we did not include trainees or individuals who left academic pathways, limiting our understanding of how ethics education is enacted and received. Future research should examine classroom practices and team-based research environments to better identify effective approaches to teaching ethical digital health research.

### Conclusion

Our study demonstrates that ethics education and learning emerge through the interaction of formal coursework and practice-based training, each cultivating distinct capabilities needed to navigate data-intensive and collaborative research environments. While these complementary pathways help prepare students to recognize ethical issues, apply principles in context, anticipate emerging risks, and communicate across disciplines, access to experiential ethics training remains uneven. Strengthening ethics education will therefore require expanding structured opportunities for early research engagement, fostering communities of practice among educators and researchers, and developing mechanisms that translate tacit ethical reasoning into shareable resources. Designing training environments that integrate ethical reflection into everyday research practice may better equip the next generation of digital health researchers to responsibly shape the sociotechnical health systems they both study and build.

## Appendix

Multimedia Appendix 1 Readings to support themes identified in the Results section.

## Abbreviations

AI: artificial intelligence
EQUATOR: Enhancing the Quality and Transparency of Health Research
HCD: human-centered design
IRB: institutional review board
SRQR: Standards for Reporting Qualitative Research

## Data Availability

The datasets generated or analyzed during this study are available from the corresponding author on reasonable request.
