# Supplementary material for: Educating Students About Digital Health Research Ethics: Curricula Review and Expert Interview Study
Source: J Med Internet Res. 2026 Mar 26;28:e82861. doi: 10.2196/82861 (PMC13066782; doi:10.2196/82861)
Supplement: Multimedia Appendix 1 [file jmir_v28i1e82861_app1.docx]

# Appendix A. Example of Course References Cited in Results Section

## Theme 1. Broad Understanding

- Integrity in Scientific Research Video Series. American Association for the Advancement of Science (AAAS. 2026. Available from: https://www.aaas.org/resources/integrated-public-use-microdata-series-ipums [accessed Mar 10, 2026] [37]
- Jones CP. Confronting Institutionalized Racism. Phylon (1960-) Clark Atlanta University; 2002;50(1/2):7–22. doi: 10.2307/4149999 [38]
- Ess C. Digital Media Ethics. Oxford Research Encyclopedia of Communication 2017. doi: 10.1093/acrefore/9780190228613.013.508ISBN:978-0-19-022861-3 [39]
- Responsible Conduct of Research Training. NIH Office of Intramural Research. Available from: https://oir.nih.gov/sourcebook/ethical-conduct/responsible-conduct-research-training [accessed Mar 10, 2026] [40]

## Theme 2. Applied Ethics

- Awad E, Dsouza S, Kim R, Schulz J, Henrich J, Shariff A, Bonnefon J-F, Rahwan I. The Moral Machine experiment. Nature Nature Publishing Group; 2018 Nov;563(7729):59–64. doi: 10.1038/s41586-018-0637-6 [41]
- Kolata G. When Doctors Use a Chatbot to Improve Their Bedside Manner. The New York Times 2023 Jun 12; Available from: https://www.nytimes.com/2023/06/12/health/doctors-chatgpt-artificial-intelligence.html [accessed Mar 11, 2026] [42]
- Obermeyer Z, Powers B, Vogeli C, Mullainathan S. Dissecting racial bias in an algorithm used to manage the health of populations. Science New York, N.Y.; 2019 Oct 25;366(6464):447–453. PMID:31649194 [43]
- Waldman AE. Cognitive biases, dark patterns, and the ‘privacy paradox.’ Current Opinion in Psychology 2020 Feb 1;31:105–109. doi: 10.1016/j.copsyc.2019.08.025 [44]

## Theme 3. Research in Context

- Conklin AWm. Principles of Computer Security: CompTIA Security+ and Beyond. 5th ed. Available from: https://booksrun.com/9781260026016-principles-of-computer-security-comptia-security-and-beyond-fifth-edition [accessed Mar 11, 2026]ISBN:978-1-260-02601-6 [71]
- Foxwell HJ. Creating Good Data: A Guide to Dataset Structure and Data Representation. Apress; 2020. Available from: https://www.oreilly.com/library/view/creating-good-data/9781484261033/ [accessed Mar 11, 2026]ISBN:978-1-4842-6103-3 [45]
- James G, Witten D, Hastie T, Tibshirani R. An Introduction to Statistical Learning: with Applications in R. 2nd ed. New York, NY: Springer US; 2021. doi: 10.1007/978-1-0716-1418-1ISBN:978-1-07-161417-4 [46]
- O’Neil C. The Ethical Data Scientist. Slate 2016 Feb 4; Available from: https://slate.com/technology/2016/02/how-to-bring-better-ethics-to-data-science.html [accessed Mar 11, 2026] [47]

## Theme 4. Ethics in Technology Design

- Dix A. Human–computer interaction: A stable discipline, a nascent science, and the growth of the long tail. Interact Comput 2010 Jan 1;22(1):13–27. doi: 10.1016/j.intcom.2009.11.007 [48]
- Garrett JJ. The Elements of User Experience: User-Centered Design for the Web and Beyond. 2nd ed. Berkeley, CA: New Riders; 2011. ISBN:978-0-321-68368-7 [49]
- Horton S, Quesenbery W. A Web for Everyone: Designing Accessible User Experiences. Rosenfeld Media; 2014. ISBN:978-1-933820-39-2 [50]
- Lazar J, Goldstein DF, Taylor A. Ensuring Digital Accessibility through Process and Policy. 1st ed. San Francisco, CA, USA: Morgan Kaufmann Publishers Inc.; 2015. ISBN:978-0-12-800646-7 [51]

## Theme 5. Anticipating the Future

- Barocas S, Selbst AD. Big Data’s Disparate Impact. Calif L Rev 2016; doi: 10.15779/Z38BG31 [52]
- Calo R. Artificial Intelligence Policy: A Primer and Roadmap. Rochester, NY: Social Science Research Network; 2017. doi: 10.2139/ssrn.3015350 [53]
- Friedler SA, Scheidegger C, Venkatasubramanian S. The (Im)possibility of fairness: different value systems require different mechanisms for fair decision making. Commun ACM 2021 Mar 22;64(4):136–143. doi: 10.1145/3433949 [54]
- Kirkpatrick N. Making a living in the toxic world of discarded electronics. The Washington Post 2015 Apr 17; Available from: https://www.washingtonpost.com/news/in-sight/wp/2015/04/15/the-children-who-make-a-living-in-the-toxic-world-of-discarded-electronics/ [accessed Mar 11, 2026] [55]
- Noble SU. Algorithms of oppression: How search engines reinforce racism. New York, NY, US: New York University Press; 2018. p. xv, 229. ISBN:978-1-4798-3724-3 [56]
- Rotman D. The disparity between the rich and everyone else is larger than ever in the United States and increasing in much of Europe. Why? MIT Technology Review 2014 Oct 21;(11/2014). Available from: https://www.technologyreview.com/2014/10/21/170679/technology-and-inequality/ [accessed Mar 11, 2026] [57]
- Shaw J. Confronting pitfalls of machine learning, artificial intelligence | Harvard Magazine. 2018 Dec 6; Available from: https://www.harvardmagazine.com/2018/12/artificial-intelligence-limitations [accessed Mar 11, 2026] [58]
- Wallach W, Allen C. Moral Machines: Teaching Robots Right from Wrong. Oxford University Press; 2009. doi: 10.1093/acprof:oso/9780195374049.001.0001ISBN:978-0-19-537404-9 [59]
- Winner L. Trust and terror: the vulnerability of complex socio‐technical systems. Science as Culture Routledge; 2004 Jun 1;13(2):155–172. doi: 10.1080/0950543042000226594 [60]

## Theme 6. Communication Practice

- Dainow DB. SIENNA D6.3: Methods for translating ethical analysis into instruments for the ethical development and deployment of emerging technologies. 2021 Oct 1; doi: 10.5281/ZENODO.5541538 [61]
- Niki A. How to Nail the Pitch: 17 Storytelling Tips for Startups. The Founder Institute. 2024. Available from: https://FI.co/insight/how-to-nail-the-pitch-17-storytelling-tips-for-startups [accessed Mar 11, 2026] [62]
- Michaelson HB. Creative Aspects of Engineering Writing. IRE Transactions on Engineering Writing and Speech 1961 Jan 1; doi: 10.1109/TEWS.1961.4322737 [63]
- Tebeaux E, Dragga S. The Essentials of Technical Communication. 5th ed. Oxford University Press; 2020. Available from: https://global.oup.com/ushe/product/the-essentials-of-technical-communication-9780197539200 [accessed Mar 11, 2026] ISBN:978-0-19-753920-0 [64]
- Trimble J. Writing with Style: Conversations on the Art of Writing. 3rd ed. Boston Munich: Pearson; 2010. ISBN:978-0-205-02880-1 [65]

## Theme 7. Professional Responsibility

- Gotterbarn D, Brinkman B, Flick C, Kirkpatrick MS, Miller K, Varansky K, Wolf MJ. ACM Code of Ethics and Professional Conduct. Association for Computing Machinery. 2018. Available from: https://www.acm.org/code-of-ethics [accessed Mar 11, 2026] [66]
- Association of American Medical Colleges. Compact Between Postdoctoral Appointees and Their Mentors. Association of American Medical Colleges; 2017. Available from: https://www.aamc.org/about-us/mission-areas/biomedical-research/post-doc-compact [accessed Mar 11, 2026] [67]
- Davis M. Thinking Like an Engineer: Studies in the Ethics of a Profession. 1st ed. New York: Oxford University Press; 1998. ISBN:978-0-19-512051-6 [68]
- Hill LA. Becoming a Manager: How New Managers Master the Challenges of Leadership. Boston, Mass: Harvard Business Review Press; 2003. ISBN:978-1-59139-182-1 [69]
- National Academies of Sciences, Engineering, and Medicine; Policy and Global Affairs; Board on Higher Education and Workforce; Committee on Effective Mentoring in STEMM. The Science of Effective Mentorship in STEMM. Dahlberg ML, Byars-Winston A, editors. Washington (DC): National Academies Press (US); 2019. PMID:31958221ISBN:978-0-309-49729-9 [70]
